# Supplementary material for: Effects of School-Based Exercise Program on Obesity and Physical Fitness of Urban Youth: A Quasi-Experiment
Source: Healthcare (Basel). 2021 Mar 22;9(3):358. doi: 10.3390/healthcare9030358 (PMC8004690; doi:10.3390/healthcare9030358)
Supplement: Supplementary file 1 [file healthcare-09-00358-s001.pdf]

## PAPS: 5 Health Factors (12 elective methods)

<Cardiopulmonary Evaluation>

① Shuttle-run (PACER)

② Distance runs or walks

③ Step tests

<Flexibility>

④ Sit-and-reach

⑤ Whole body flexibility

<Muscular Strength and Endurance>

⑥ Push-ups

⑦ Sit-up

⑧ Grip strength

<Quickness>

⑨ 50m run

⑩ Standing long jumping

<Body Composition>

⑪ Body Mass Index

⑫ %Fat

## [PACER] (unit: repetition) *4th~6th: 15m, 7th~12th: 20m

Male

| Grade | Very Poor (5등급) | Poor (4등급) | Fair (3등급) | Good (2등급) | Excellent (1등급) |
|---|---|---|---|---|---|
| 4th | 19~25 | 26~44 | 45~68 | 69~95 | 96~103 |
| 5th | 22~28 | 29~49 | 50~72 | 73~99 | 100~107 |
| 6th | 22~31 | 32~53 | 54~77 | 78~103 | 104~112 |
| 7th | 15~19 | 20~35 | 36~49 | 50~63 | 64~73 |
| 8th | 15~21 | 22~37 | 38~51 | 52~65 | 66~75 |
| 9th | 16~23 | 24~39 | 40~53 | 54~67 | 68~79 |
| 10th | 16~25 | 26~41 | 42~55 | 56~69 | 70~80 |
| 11th | 16~27 | 28~43 | 44~57 | 58~71 | 72~81 |
| 12th | 18~29 | 30~45 | 46~59 | 60~73 | 74~82 |

Female

| Grade | Very Poor (5등급) | Poor (4등급) | Fair (3등급) | Good (2등급) | Excellent (1등급) |
|---|---|---|---|---|---|
| 4th | 16~20 | 21~39 | 40~56 | 57~76 | 77~100 |
| 5th | 18~22 | 23~44 | 45~62 | 63~84 | 85~104 |
| 6th | 20~24 | 25~49 | 50~68 | 69~92 | 93~112 |
| 7th | 10~13 | 14~18 | 19~24 | 25~34 | 35~70 |
| 8th | 10~14 | 15~20 | 21~28 | 29~39 | 40~75 |
| 9th | 12~15 | 16~22 | 23~32 | 33~44 | 45~80 |
| 10th | 13~16 | 17~24 | 25~36 | 37~49 | 50~85 |
| 11th | 14~17 | 18~26 | 27~40 | 41~54 | 55~90 |
| 12th | 14~17 | 18~26 | 27~40 | 41~54 | 55~90 |

## [Distance runs or walks] (unit: sec)

*5$^{th}$~6$^{th}$: 1000m, Female: 1200m, Male: 1600m

Male

| Grade | Very Poor (5등급) | Poor (4등급) | Fair (3등급) | Good (2등급) | Excellent (1등급) |
|---|---|---|---|---|---|
| 5th | 480~640 | 410~479 | 325~409 | 282~324 | 268~281 |
| 6th | 450~587 | 380~449 | 315~379 | 251~314 | 243~250 |
| 7th | 700~791 | 600~699 | 503~599 | 426~502 | 400~425 |
| 8th | 680~774 | 584~679 | 488~583 | 417~487 | 380~416 |
| 9th | 660~761 | 568~659 | 473~567 | 408~472 | 370~407 |

| | | | | | |
|---|---|---|---|---|---|
| 10th | 640~755 | 552~639 | 458~551 | 399~457 | 370~398 |
| 11th | 620~721 | 536~619 | 443~535 | 390~442 | 370~389 |
| 12th | 600~701 | 520~599 | 428~519 | 381~427 | 362~380 |

Female

| Grade | Very Poor (5등급) | Poor (4등급) | Fair (3등급) | Good (2등급) | Excellent (1등급) |
|---|---|---|---|---|---|
| 5th | 502~640 | 442~501 | 360~441 | 300~359 | 268~299 |
| 6th | 480~587 | 430~479 | 354~429 | 300~353 | 243~299 |
| 7th | 609~661 | 518~608 | 443~517 | 380~442 | 355~379 |
| 8th | 609~661 | 518~608 | 443~517 | 380~442 | 355~379 |
| 9th | 609~661 | 518~608 | 443~517 | 380~442 | 355~379 |
| 10th | 609~661 | 518~608 | 443~517 | 380~442 | 365~379 |
| 11th | 609~661 | 518~608 | 443~517 | 380~442 | 365~379 |
| 12th | 609~661 | 518~608 | 443~517 | 380~442 | 365~379 |

## [Step test] (unit: PEI)

| Grade | Very Poor (5등급) | Poor (4등급) | Fair (3등급) | Good (2등급) | Excellent (1등급) |
|---|---|---|---|---|---|
| 전 학년 | 44.0~46.9 | 47.0~51.9 | 52.0~61.9 | 62.0~75.9 | 76.0~95.0 |

[Whole body flexibility] (unit: score)

| Grade | Very Poor (5등급) | Poor (4등급) | Fair (3등급) | Good (2등급) | Excellent (1등급) |
|---|---|---|---|---|---|
| All Grades | <4 | 5 | 6 | 7 | 8 |

## [Sit-and-reach] (unit: cm)

Male

| Grade | Very Poor (5등급) | Poor (4등급) | Fair (3등급) | Good (2등급) | Excellent (1등급) |
|---|---|---|---|---|---|
| 5th | -5.1~-4.1 | -4.0~0.9 | 1.0~4.9 | 5.0~7.9 | 8.0~18.0 |
| 6th | ~5.1~-4.1 | -4.0~0.9 | 1.0~4.9 | 5.0~7.9 | 8.0~18.0 |
| 7th | -5.1~-4.1 | -4.0~1.9 | 2.0~5.9 | 6.0~9.9 | 10.0~25.0 |
| 8th | -5.1~-4.1 | -4.0~1.9 | 2.0~6.9 | 7.0~9.9 | 10.0~25.0 |
| 9th | -5.1~-3.1 | -3.0~2.5 | 2.6~6.9 | 7.0~9.9 | 10.0~25.0 |
| 10th | -3.1~-2.1 | -2.0~3.9 | 4.0~8.9 | 9.0~12.9 | 13.0~28.0 |
| 11th | -3.1~0.0 | 0.1~4.9 | 5.0~10.9 | 11.0~15.9 | 16.0~28.0 |
| 12th | -3.1~0.0 | 0.1~5.9 | 6.0~10.9 | 11.0~15.9 | 16.0~28.0 |

Female

| Grade | Very Poor (5등급) | Poor (4등급) | Fair (3등급) | Good (2등급) | Excellent (1등급) |
|---|---|---|---|---|---|
| 5th | -0.1~0.9 | 1.0~4.9 | 5.0~6.9 | 7.0~9.9 | 10.0~22.0 |
| 6th | -0.1~1.9 | 2.0~4.9 | 5.0~9.9 | 10.0~13.9 | 14.0~26.0 |
| 7th | -0.1~1.9 | 2.0~7.9 | 8.0~10.9 | 11.0~14.9 | 15.0~28.0 |
| 8th | -0.1~1.9 | 2.0~7.9 | 8.0~10.9 | 11.0~14.9 | 15.0~28.0 |
| 9th | -0.1~1.9 | 2.0~7.9 | 8.0~10.9 | 11.0~15.9 | 16.0~28.0 |
| 10th | -0.1~1.9 | 2.0~7.9 | 8.0~10.9 | 11.0~15.9 | 16.0~28.0 |

| | | | | | |
|---|---|---|---|---|---|
| 11th | -0.1~4.9 | 5.0~8.9 | 9.0~11.9 | 12.0~16.9 | 17.0~28.0 |
| 12th | -0.1~4.9 | 5.0~8.9 | 9.0~11.9 | 12.0~16.9 | 17.0~28.0 |

## [Push-ups] (unit: repetitions)

Male

| Grade | Very Poor (5등급) | Poor (4등급) | Fair (3등급) | Good (2등급) | Excellent (1등급) |
|---|---|---|---|---|---|
| 7th | 0~3 | 4~11 | 12~24 | 25~33 | 34~41 |
| 8th | 0~3 | 4~11 | 12~24 | 25~33 | 34~41 |
| 9th | 0~3 | 4~13 | 14~24 | 25~33 | 34~41 |
| 10th | 2~6 | 7~15 | 16~29 | 30~45 | 46~50 |
| 11th | 7~10 | 11~24 | 25~41 | 42~49 | 50~54 |
| 12th | 7~16 | 17~29 | 30~45 | 46~55 | 56~62 |

Female

| Grade | Very Poor (5등급) | Poor (4등급) | Fair (3등급) | Good (2등급) | Excellent (1등급) |
|---|---|---|---|---|---|
| 7th | 0~5 | 6~13 | 14~23 | 24~44 | 45~50 |
| 8th | 0~5 | 6~13 | 14~23 | 24~39 | 40~44 |

| | | | | | |
|---|---|---|---|---|---|
| 9th | 0~5 | 6~13 | 14~23 | 24~39 | 40~44 |
| 10th | 0~5 | 6~13 | 14~23 | 24~39 | 40~44 |
| 11th | 0~8 | 9~17 | 18~29 | 30~39 | 40~44 |
| 12th | 0~8 | 9~17 | 18~29 | 30~39 | 40~44 |

## [Grip strength] (unit: kg)

Male

| Grade | Very Poor (5등급) | Poor (4등급) | Fair (3등급) | Good (2등급) | Excellent (1등급) |
|---|---|---|---|---|---|
| 4th | 16~20 | 21~39 | 40~56 | 57~76 | 77~100 |
| 5th | 18~22 | 23~44 | 45~62 | 63~84 | 85~104 |
| 6th | 20~24 | 25~49 | 50~68 | 69~92 | 93~112 |
| 7th | 10~13 | 14~18 | 19~24 | 25~34 | 35~70 |
| 8th | 10~14 | 15~20 | 21~28 | 29~39 | 40~75 |

| | | | | | |
|---|---|---|---|---|---|
| 9th | 12~15 | 16~22 | 23~32 | 33~44 | 45~80 |
| 10th | 13~16 | 17~24 | 25~36 | 37~49 | 50~85 |
| 11th | 14~17 | 18~26 | 27~40 | 41~54 | 55~90 |
| 12th | 14~17 | 18~26 | 27~40 | 41~54 | 55~90 |

Female

| Grade | Very Poor (5등급) | Poor (4등급) | Fair (3등급) | Good (2등급) | Excellent (1등급) |
|---|---|---|---|---|---|
| 4th | 16~20 | 21~39 | 40~56 | 57~76 | 77~100 |
| 5th | 18~22 | 23~44 | 45~62 | 63~84 | 85~104 |
| 6th | 20~24 | 25~49 | 50~68 | 69~92 | 93~112 |
| 7th | 10~13 | 14~18 | 19~24 | 25~34 | 35~70 |
| 8th | 10~14 | 15~20 | 21~28 | 29~39 | 40~75 |
| 9th | 12~15 | 16~22 | 23~32 | 33~44 | 45~80 |
| 10th | 13~16 | 17~24 | 25~36 | 37~49 | 50~85 |
| 11th | 14~17 | 18~26 | 27~40 | 41~54 | 55~90 |
| 12th | 14~17 | 18~26 | 27~40 | 41~54 | 55~90 |

## [50m run] (unit: sec)

Male

| Grade | Very Poor (5등급) | Poor (4등급) | Fair (3등급) | Good (2등급) | Excellent (1등급) |
|---|---|---|---|---|---|
| 4th | 13.21~16.01 | 10.51~13.20 | 9.71~10.50 | 8.81~9.70 | 8.70~8.80 |
| 5th | 13.21~15.56 | 10.21~13.20 | 9.41~10.20 | 8.51~9.40 | 8.30~8.50 |
| 6th | 12.51~15.51 | 10.01~12.50 | 9.11~10.00 | 8.11~9.10 | 7.77~8.10 |
| 7th | 11.51~12.51 | 9.31~11.50 | 8.41~9.30 | 7.51~8.40 | 7.40~7.50 |
| 8th | 11.51~12.41 | 9.01~11.50 | 8.21~9.00 | 7.31~8.20 | 7.11~7.30 |
| 9th | 11.01~12.38 | 8.51~11.00 | 7.81~8.50 | 7.01~7.80 | 6.80~7.00 |
| 10th | 10.01~10.74 | 8.11~10.00 | 7.61~8.10 | 7.01~7.60 | 6.80~7.00 |
| 11th | 9.51~9.71 | 7.91~9.50 | 7.51~7.90 | 6.71~7.50 | 6.50~6.70 |
| 12th | 8.71~9.61 | 7.91~8.70 | 7.51~7.90 | 6.71~7.50 | 6.40~6.70 |

Female

| Grade | Very Poor (5등급) | Poor (4등급) | Fair (3등급) | Good (2등급) | Excellent (1등급) |
|---|---|---|---|---|---|
| 4th | 13.31~18.42 | 11.01~13.30 | 10.41~11.00 | 9.41~10.40 | 9.30~9.40 |
| 5th | 13.01~15.91 | 10.71~13.00 | 9.91~10.70 | 8.91~9.90 | 8.73~8.90 |
| 6th | 12.91~15.01 | 10.71~12.90 | 9.81~10.70 | 8.91~9.80 | 8.66~8.90 |
| 7th | 12.21~13.51 | 10.51~12.20 | 9.81~10.50 | 8.81~9.80 | 8.60~8.80 |
| 8th | 12.21~13.51 | 10.51~12.20 | 9.81~10.50 | 8.81~9.80 | 8.60~8.80 |
| 9th | 12.21~13.51 | 10.51~12.20 | 9.81~10.50 | 8.81~9.80 | 8.60~8.80 |
| 10th | 12.21~13.51 | 10.51~12.20 | 9.81~10.50 | 8.81~9.80 | 8.60~8.80 |
| 11th | 12.21~13.51 | 10.51~12.20 | 9.51~10.50 | 8.81~9.50 | 8.60~8.80 |

| | | | | | |
|---|---|---|---|---|---|
| 12th | 12.21~13.51 | 10.51~12.20 | 9.51~10.50 | 8.81~9.50 | 8.60~8.80 |

## [Standing long jumping] (unit: cm)

Male

| Grade | Very Poor (5등급) | Poor (4등급) | Fair (3등급) | Good (2등급) | Excellent (1등급) |
|---|---|---|---|---|---|
| 4th | 98.2~100 | 100.1~130 | 130.1~149 | 149.1~170 | 170.1~179.4 |
| 5th | 105.6~111 | 111.1~141 | 141.1~159 | 159.1~180 | 180.1~187.4 |
| 6th | 112.0~122 | 122.1~148 | 148.1~167 | 167.1~200 | 200.1~204.7 |
| 7th | 122.9~131 | 131.1~159 | 159.1~177 | 177.1~211 | 211.1~219.7 |
| 8th | 129.9~136 | 136.1~169 | 169.1~187 | 187.1~218 | 218.1~229.3 |
| 9th | 141.4~145 | 145.1~180 | 180.1~201 | 201.1~238 | 238.1~244.0 |
| 10th | 139.9~160 | 160.1~195 | 195.1~216 | 216.1~255 | 255.1~260.2 |
| 11th | 162.9~177 | 177.1~212 | 212.1~228 | 228.1~258 | 258.1~270.0 |
| 12th | 169.9~185 | 185.1~221 | 221.1~243 | 243.1~264 | 264.1~277.1 |

Female

| Grade | Very Poor (5등급) | Poor (4등급) | Fair (3등급) | Good (2등급) | Excellent (1등급) |
|---|---|---|---|---|---|
| 4th | 87.4~97 | 97.1~119 | 119.1~135 | 135.1~161 | 161.1~165.5 |
| 5th | 89.3~100 | 100.1~123 | 123.1~139 | 139.1~170 | 170.1~175.0 |

| | | | | | |
|---|---|---|---|---|---|
| 6th | 89.9~100 | 100.1~127 | 127.1~144 | 144.1~175 | 175.1~177.8 |
| 7th | 89.9~100 | 100.1~127 | 127.1~144 | 144.1~175 | 175.1~180.5 |
| 8th | 93.9~100 | 100.1~127 | 127.1~145 | 145.1~183 | 183.1~198.0 |
| 9th | 94.9~100 | 100.1~127 | 127.1~145 | 145.1~183 | 183.1~198.0 |
| 10th | 94.9~100 | 100.1~139 | 139.1~159 | 159.1~186 | 186.1~200.0 |
| 11th | 94.9~100 | 100.1~139 | 139.1~159 | 159.1~186 | 186.1~200.0 |
| 12th | 94.9~100 | 100.1~139 | 139.1~159 | 159.1~186 | 186.1~200.0 |

## [Body Mass Index] (unit: $kg/m^2$)

Male

| Grade | Lean | Normal | Overweight | Mild Obesity | Severe Obesity |
|---|---|---|---|---|---|
| 4th | <14.0 | 14.1~20.1 | 20.2~22.3 | 22.4~32.3 | 32.4이상 |
| 5th | <14.3 | 14.4~20.9 | 21.0~23.3 | 23.4~33.3 | 33.4이상 |
| 6th | <14.9 | 15.0~21.7 | 21.8~24.0 | 24.1~34.0 | 34.1이상 |
| 7th | <15.4 | 15.5~22.6 | 22.7~24.7 | 24.8~34.7 | 34.8이상 |
| 8th | <16.1 | 16.2~23.1 | 23.2~25.6 | 25.7~35.6 | 35.7이상 |
| 9th | <16.7 | 16.8~23.7 | 23.8~25.9 | 26.0~35.9 | 36.0이상 |
| 10th | <17.3 | 17.4~23.8 | 23.9~26.0 | 26.1~36.0 | 36.1이상 |
| 11th | <17.6 | 17.7~23.7 | 23.8~25.9 | 26.0~35.9 | 36.0이상 |
| 12th | <18.0 | 18.1~23.7 | 23.8~25.5 | 25.6~35.5 | 35.6이상 |

Female

| Grade | Lean | Normal | Overweight | Mild Obesity | Severe Obesity |
|---|---|---|---|---|---|
| 4th | <14.3 | 14.4~21.1 | 21.2~23.7 | 23.8~33.8 | 33.9< |
| 5th | <14.6 | 14.7~21.7 | 21.8~24.4 | 24.5~34.5 | 34.6< |
| 6th | <15.0 | 15.1~22.3 | 22.4~25.1 | 25.2~35.2 | 35.3< |
| 7th | <15.5 | 15.6~22.9 | 23.0~25.7 | 25.8~35.8 | 35.9< |
| 8th | <15.9 | 16.0~23.3 | 23.4~26.2 | 26.3~36.3 | 36.4< |
| 9th | <16.5 | 16.6~23.7 | 23.8~26.6 | 26.7~36.7 | 36.8< |
| 10th | <16.9 | 17.0~24.1 | 24.2~26.8 | 26.9~36.9 | 37.0< |
| 11th | <17.4 | 17.5~24.3 | 24.4~26.9 | 27.0~37.0 | 37.1< |
| 12th | <17.9 | 18.0~24.5 | 24.6~26.8 | 26.9~36.9 | 37.0< |

## [%Fat] (unit: %)

| | Lean | Normal | Overweight | Mild Obesity | Severe Obesity |
|---|---|---|---|---|---|
| Male | <11.9 | 12 | 15 | 25 | 33< |
| Female | <14.9 | 15 | 27 | 32 | 40< |
